# Inversion study on elastic–plastic material parameters of red sandstone in uniaxial compression test

**DOI:** 10.1038/s41598-023-49126-w

**Published:** 2023-12-09

**Authors:** Jianing Wu, Xiaobin Yang, Yimin Song, Shun Liu, Shihao Li, Jiawei Liu

**Affiliations:** 1 School of Emergency Management and Safety Engineering, China University of Mining and Technology-Beijing, Beijing, 100083 China; 2 School of Civil Engineering, North China University of Technology, Beijing, 100144 China

**Keywords:** Theory and computation, Civil engineering

## Abstract

In order to obtain the real material parameters of heterogeneous rock, the material parameters of red sandstone specimens under uniaxial compression tests are inverted based on the Digital Image Correlation (DIC) method and the Finite Element Model Updating (FEMU) method. The DIC method is employed to calculate the displacement field of red sandstone specimens during uniaxial compression loading. Concurrently, a uniaxial compression elastic–plastic finite element numerical model with non-uniform material parameters is developed based on the FEMU method. The model adopts the Mohr–Coulomb yield criterion and adjusts the boundary conditions in real-time to maintain consistency with the test. The vertical displacement field of the numerical model is juxtaposed with that of the test to construct the objective function. Optimization is achieved using the Artificial Fish Swarm algorithm, which enables the acquisition of the non-uniform distribution and evolution process of the material parameters of specimens at different loading moments. The results indicate that this method can spatially obtain the non-uniform distribution field of material parameters and temporally track the evolution of material parameters during the loading process. This research lays a solid foundation for enhancing the accuracy of intelligent coal mining and dynamic disaster monitoring and early warning in coal mines.

## Introduction

Rock material parameters serve as essential foundational data for theoretical analysis and numerical computation, playing a significant role in research fields such as dynamic disaster monitoring and intelligent coal mining^1–4^. The complexity of rocks, which are composed of various minerals, influenced by geostress changes, structural geology, weathering effects, and contain numerous joints and fractures, results in pronounced spatial non-uniformity in their material parameters^5–8^. Current research often assigns uniform rock material parameters from a macroscopic perspective or assumes that these parameters follow a specific probability distribution model, which is then input into numerical simulation software. However, such computational outcomes do not accurately represent the true mechanical response of rock materials. Conversely, the micro-fractures within the rock undergo rapid development and alteration when exposed to different external loads, leading to changes in the overall material properties of the rock in response to load variation. Therefore, performing inverse analysis on rock material parameters and conducting research on their non-uniformity and evolutionary laws are critical foundations for enhancing the accuracy of intelligent coal mining and dynamic disaster monitoring systems. This also enables the real-time monitoring of multi-scale and multi-index information, holding substantial theoretical and engineering value.

Research on rock material parameters often employs numerical simulation methods, which can be categorized into continuous methods, discontinuous methods, and coupled methods. Continuous methods encompass the Finite Element Method (FEM) and the Finite Difference Method (FDM), while discontinuous methods consist of the Discrete Element Method (DEM). Coupled methods include the Finite-Discrete Element Method (FDEM) and others. Techniques for characterizing the non-uniformity of rock materials involve the Digital Image Correlation (DIC) and the assignment of specific probability distribution parameters. Notably, the Digital Image Correlation is frequently combined with software such as ABAQUS, FLAC, PFC, and UDEC to conduct numerical simulations and analyze rock damage characteristics and damage evolution^9,10^. The method of assigning specific probability distribution parameters often relies on the Realistic Failure Process Analysis (RFPA) model or the Elasto-Plastic Cellular Automaton (EPCA) model, assuming that material parameters follow Weibull distribution, normal distribution, lognormal distribution, and so on, and establishing a damage and failure model for non-uniform material parameters^11,12^. Regarding research on material parameter inversion, the primary methods include the Virtual Fields Method (VFM) and Finite Element Model Updating (FEMU). The VFM method utilizes full-field deformation measurement data, expresses the structural strain energy through the material constitutive relationship and the virtual displacement field that satisfies displacement boundary conditions and continuity conditions, and obtains the relationship between the measured deformation, load, and unknown material parameters in the constitutive model according to the virtual work principle^13^. Guo et al.^14^ calculated the Young's modulus and Poisson's ratio of graphite materials. Jiang et al.^15^ used the VFM method to invert the elastic–plastic parameters of laser-welded joints. Zhang et al.^16^ employed the 3D Digital Image Correlation (DIC) to characterize the heterogeneous strain field of Carbon Fiber Reinforced Plastic (CFRP) specimens and derived the specimen's Young's modulus, shear modulus, and Poisson's ratio using the VFM method. The principle of the FEMU method is to compare data obtained from tests with the results of finite element model calculations. The parameters of the finite element model are continuously revised to make the simulated values closely match the experimental values, thereby obtaining a finite element model that more accurately reflects the actual structural characteristics^17,18^. Currently, the application of the FEMU method in rock materials is less common. Yin et al.^19^ proposed a rock elastic parameter inversion method based on DIC and FEM, which obtained the fields of Young's modulus and Poisson's ratio under complex stress states. Song et al.^20^ and Wu et al.^21^ conducted inversions for the mechanical parameters of red sandstone and geotechnical analogous model materials, separately based on DIC and FEMU. Cong et al.^22^ developed a method using FEMU for determining sensitive complex material parameters associated with piezoceramic plates. Liu et al.^23^ conducted a study on the damage characteristics of IG11 graphite material under complex stress states based on FEMU. The FEMU method is widely used in the research of parameter identification of various materials^24–26^.

In the aforementioned studies, the rock material parameters are predicated on a certain assumption of a probability distribution function, which deviates from the actual parameter distribution. Additionally, numerical models often opt for elastic constitutive equations, and there is a dearth of research into the rock material parameters and their non-uniformity that change during the loading process. To address these issues, this paper proposes a method for the inversion of elastic–plastic constitutive material parameters in the uniaxial compression test of red sandstone, based on the Digital Image Correlation (DIC) and the Finite Element Model Updating (FEMU). The displacement field of the red sandstone uniaxial compression test is considered a known quantity, and the material parameters of the red sandstone specimen are inverted. The Artificial Fish Swarm algorithm is employed for optimization and solution. This approach enables the acquisition of the non-uniform distribution field of material parameters during the test loading process from a spatial perspective, and facilitates understanding of the evolution process of material parameters with loading over time.

## Inversion method of rock material parameters

**Figure 1:**
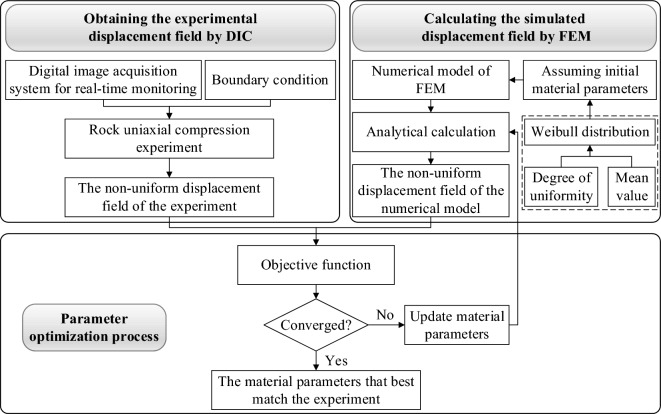
Inversion method of rock material parameters.

The implementation of the inversion method for the non-uniform distribution of rock material parameters primarily relies on two techniques: the Digital Image Correlation (DIC) and the Finite Element Model Updating (FEMU). The inversion method is segmented into three components: acquisition of the experimental displacement field via DIC, obtaining the simulated displacement field through finite element numerical analysis, and the parameter optimization process, as depicted in Fig. 1. Initially, a digital image acquisition system is employed to record the displacement field of rock specimens during the uniaxial compression test. Subsequently, based on the geometric dimensions of the specimens and the test boundary conditions, the finite element software ABAQUS is utilized to construct a uniaxial compression numerical model. Each element is assigned different material parameters according to the Weibull distribution, and finite element analysis calculations are executed. A Python script is composed to automatically output the simulated displacement field. Finally, within the MATLAB software, the experimental displacement field computed by DIC and the simulated displacement field calculated by ABAQUS are compared. The Artificial Fish Swarm Algorithm is employed to continuously update the material parameters of each element in the finite element model, minimizing the discrepancy between the experimental displacement and the simulated displacement. This process results in the acquisition of the material parameters that best correspond to the specimen.

The parameter inversion process is predicated on the joint utilization of MATLAB-Python-ABAQUS across multiple platforms. MATLAB governs the computational process, reading the experimental displacement field observed by DIC, and invoking Python scripts to read the simulated displacement field in the ABAQUS result file (.odb). It constructs an objective function by comparing the simulated displacement field with the experimental displacement field. An optimization algorithm is then employed for the iterative solution of the objective function. If the iterative process does not meet the convergence criteria, MATLAB must re-invoke the Python script to adjust the material parameters in the ABAQUS input file (.inp) and execute the ABAQUS computational routine. A Python script is subsequently invoked to read the simulated displacement field from the ABAQUS output database file (.odb).

## Inversion process of rock material parameters

### Test

**Figure 2:**
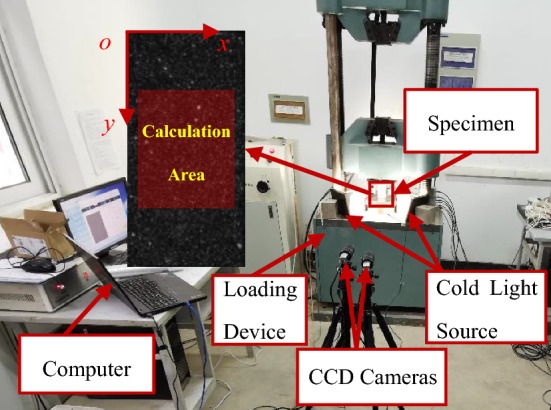
The layout of experimental system.

A red sandstone material is chosen and processed into a rectangular specimen with dimensions of 50 mm × 50 mm × 100 mm. The surface of the specimen is uniformly coated with a layer of black primer. Once the black paint is completely dry, random white spots are sprayed onto the surface, creating an artificial speckle field of white spots against a black background. The displacement field of the specimen is observed and calculated using the DIC system, which comprises a CCD camera, cold light source, and a computer. The configuration of the experimental system is illustrated in Fig. 2.

Prior to the test, all devices of the loading system and the DIC system are activated. The specimen is placed at the center of the lower pressure plate of the testing machine. The brightness of the cold light source is adjusted, along with the position, brightness, and focal length of the CCD camera, to ensure that each pixel of the speckle field on the specimen's surface is clearly displayed in the DIC system. The cold light source continuously illuminates the specimen to maintain constant brightness of the speckle field throughout the loading process. Subsequently, the loading system is synchronized with the digital image acquisition system to ensure temporal consistency between the two systems. Upon commencement of the experiment, the loading device is activated. The digital image acquisition system captures images at a rate of 5 frames per second, with an image resolution of 1600 × 1200 pixels and an object surface resolution of 0.0887 mm per pixel. During the test, the upper pressure plate of the testing machine remains stationary, while the lower pressure plate applies upward displacement at a loading rate of 0.06 mm per minute. This continues until the specimen experiences failure, at which point loading and data acquisition are terminated. Following the conclusion of the test, the acquired load data and speckle images are analyzed and processed to calculate the displacement field at different loading instants.

**Figure 3:**
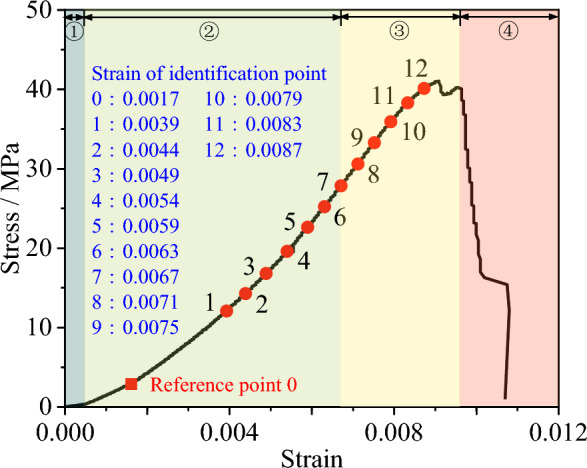
Stress–strain curve under uniaxial compression.

A total of 3825 speckle images were collected during the test. The corresponding stress–strain curve is shown in Fig. 3. The stress–strain curve under uniaxial loading of rock can be divided into the following four stages: (1) Pore fracture compaction stage, (2) Elastic deformation to microelastic fracture stable development stage (elastic stage), (3) Unstable fracture development stage (elastic–plastic stage), and (4) Post-fracture stage. During the processing of the collected speckle images, the speckle image collected at the loading time corresponding to point 0 in the figure (with the strain of 0.0017) was selected as the reference image. Points 1–12 were selected at equal strain intervals on the stress–strain curve, and the displacement field of the speckle images at the corresponding loading moments was calculated. Notably, during the compression test, due to the difference in mechanical properties between the upper and lower ends of the loading and the rock specimen, relative motion between the loading ends and the rock will be generated during the loading process, resulting in relative displacement and friction between the loading end and the rock, which is referred to as end friction. The horizontal displacement near the end is noticeably smaller than the horizontal displacement away from the end, and the farther the distance from the loading end, the smaller the influence of the end friction effect. Therefore, to reduce the influence of the end friction on the calculation result, the part with significant boundary effect should be removed from the calculation area. By referring to relevant literature^27–29^ and combining with the specific conditions of this experiment, the upper and lower ends were indented by 25%, that is, 25 mm respectively. Since there is no loading end in the horizontal direction, the left and right sides of the speckle surface were indented by 10%, that is, 5 mm. The length of the entire calculation area is 40 mm and the height is 50 mm, as shown in Fig. 2. The calculation correlation window size is 29 × 29 pixels, with a step size of 5 pixels. A speckle image contains deformation data of approximately 10,000 pixels in the calculation area.

### Numerical model

A three-dimensional finite element model of uniaxial compression is constructed in ABAQUS. The model dimensions are 50 mm × 50 mm × 100 mm, and the element properties consist of 8-node hexahedral fully integrated elements (C3D8), with an element size of 5 mm. The specimen comprises a total of 2541 nodes and 2000 elements. The parameter inversion calculation area aligns with the DIC calculation area in terms of location and size, with a length of 40 mm and a height of 50 mm, consisting of 80 elements and 99 nodes. The model adopts the Mohr–Coulomb yield criterion, the formula for which is:

1 $$\tau =\sigma tan\varphi +c$$

In Eq. (1), $\tau$ represents the shear stress on the oblique section, $\sigma$ denotes the normal stress on the oblique section, $\varphi$ is the internal friction angle with a range of 0 ≤ $\varphi$≤90°, and $c$ stands for the cohesion. Material parameters are assigned to the finite element model according to the Weibull distribution, serving as the initial values for the computation of element material parameters. This process generates the displacement field of the finite element model. The computation formula for the Weibull distribution function is:

2 $$\alpha ={\alpha }_{0}{\left[-\mathrm{ln}\left(1-f\left(\alpha \right)\right)\right]}^\frac{1}{m}$$

**Figure 4:**
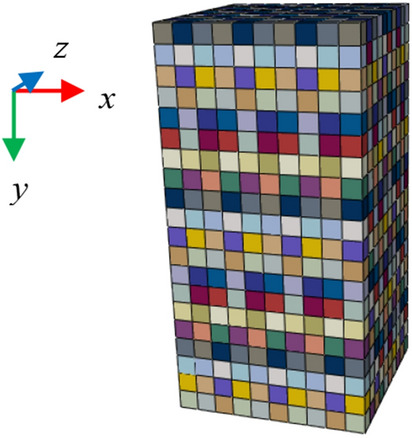
The schematic diagram of the material parameter distribution.

**Figure 5:**
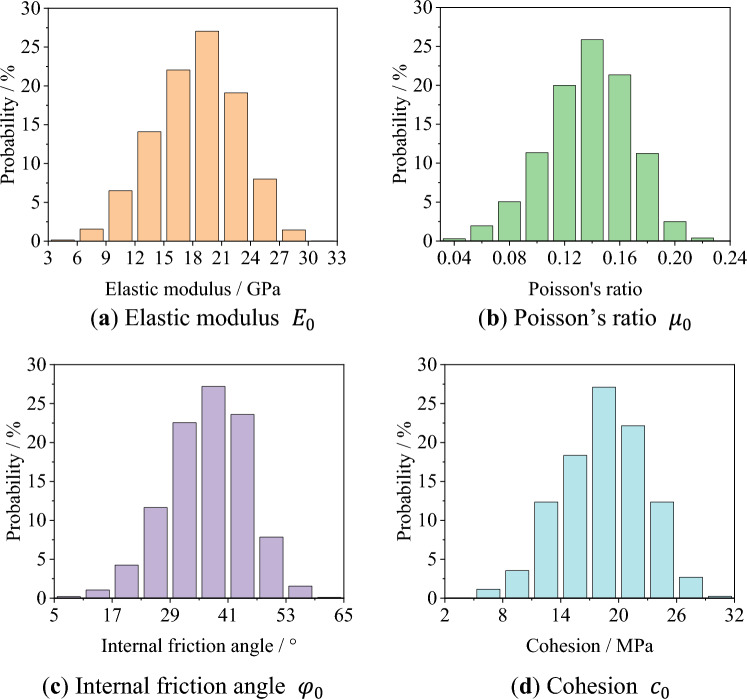
The initial distribution of ${E}_{0}$,${\mu }_{0}$,${\varphi }_{0}$ and ${c}_{0}$.

In Eq. (2), $\alpha$ denotes the material parameters of each element, while ${\alpha }_{0}$ represents the mean of material parameters, expressed as the elastic modulus ${E}_{0}$, Poisson's ratio ${\mu }_{0}$, internal friction angle ${\varphi }_{0}$, and cohesion ${c}_{0}$. The probability density function is represented by $f\left(\alpha \right)$, which is a random number between [0,1]. The shape parameter of the Weibull distribution function is denoted by *m*, which characterizes the degree of non-uniformity of the rock. The larger value of *m* indicates a more concentrated distribution of the rock material parameters, suggesting a stronger uniformity of the rock. Conversely, a smaller value of *m* implies a more dispersed distribution of the rock material parameters, indicating a stronger non-uniformity of the rock. According to relevant references^30–36^, given the Weibull distribution shape parameter *m* = 5, the mean parameters ${E}_{0}$=20 GPa, ${\mu }_{0}$=0.15, ${c}_{0}$=20 MPa, ${\varphi }_{0}$=40°. a Python script is used to assign the generated material parameters to the finite element model. Figure 4 shows the schematic diagram of the material parameter distribution, with the distribution of the elastic modulus ${E}_{0}$, Poisson's ratio ${\mu }_{0}$, internal friction angle ${\varphi }_{0}$, and cohesion ${c}_{0}$ as shown in Fig. 5. In accordance with the vertical displacement field of different loading stages in the uniaxial compression test, the boundary conditions of the finite element numerical model are adjusted in real-time to maintain consistency between the numerical model and the test. Finite element calculations are then performed on the model to obtain the displacement field of the model.

### Objective function and optimization

During the experimental loading process, the deformation of the rock is calculated using the DIC method, which directly yields the displacement field data. On the other hand, in a uniaxial compression test, axial displacement provides the most intuitive deformation data. Therefore, the vertical displacement (axial displacement) is adopted as the computation object for the objective function.

The objective function is defined as the sum of the squares of the differences between the experimental measurement displacement and numerical simulation displacement for all nodes within the computation area in the vertical direction, as shown in Eq. (3). In this equation, ${v}_{i}^{exp}$ represents the vertical displacement calculated in the experiment through DIC method, ${v}_{i}^{num}$ represents the vertical displacement calculated in the numerical simulation, and *n* represents the number of nodes in the finite element model within the computation area.

3 $$Q=\sum_{i=1}^{n}\left[{\left({v}_{i}^{num}-{v}_{i}^{exp}\right)}^{2}\right]$$

**Figure 6:**
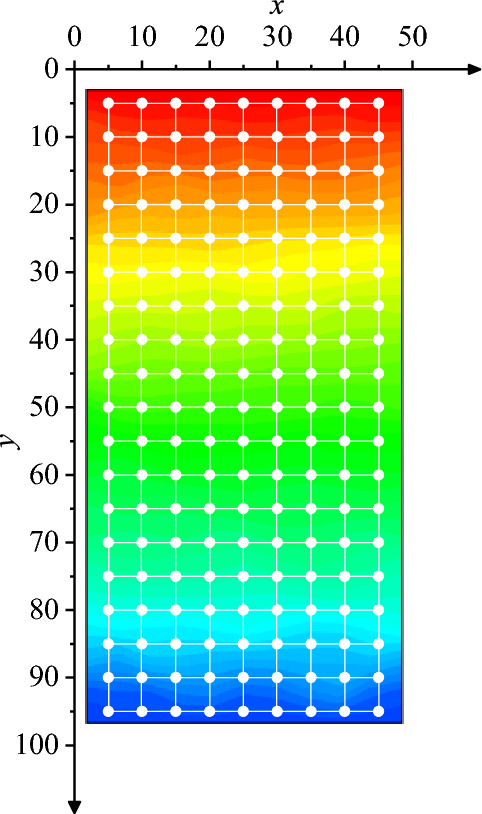
Schematic diagram of finite element model node interpolation.

To precisely compare the experimental measurement displacement field with the finite element model displacement field, the DIC computation point coordinates, the vertical displacement, and the finite element mesh node coordinates are taken as known values. Considering the finite element grid density and node coordinates, the experimental displacement field data corresponding to the finite element model node coordinates are computed using cubic spline interpolation in MATLAB. A schematic of the finite element model node interpolation is shown in Fig. 6. The white dots represent the finite element nodes, while the background cloud diagram represents the vertical displacement field cloud diagram computed using the DIC method. It should be particularly noted that according to the calculation principle of the DIC method, a pixel that is consistent with the gray level of the calculation point is searched in the half calculation correlation window around the calculation point. This pixel is considered to be the calculation point after deformation. Consequently, the calculation result will inevitably sacrifice the pixel point of half the calculation correlation window (29 pixels in the calculation), meaning that the specimen boundary will be indented by 14 pixels.

**Figure 7:**
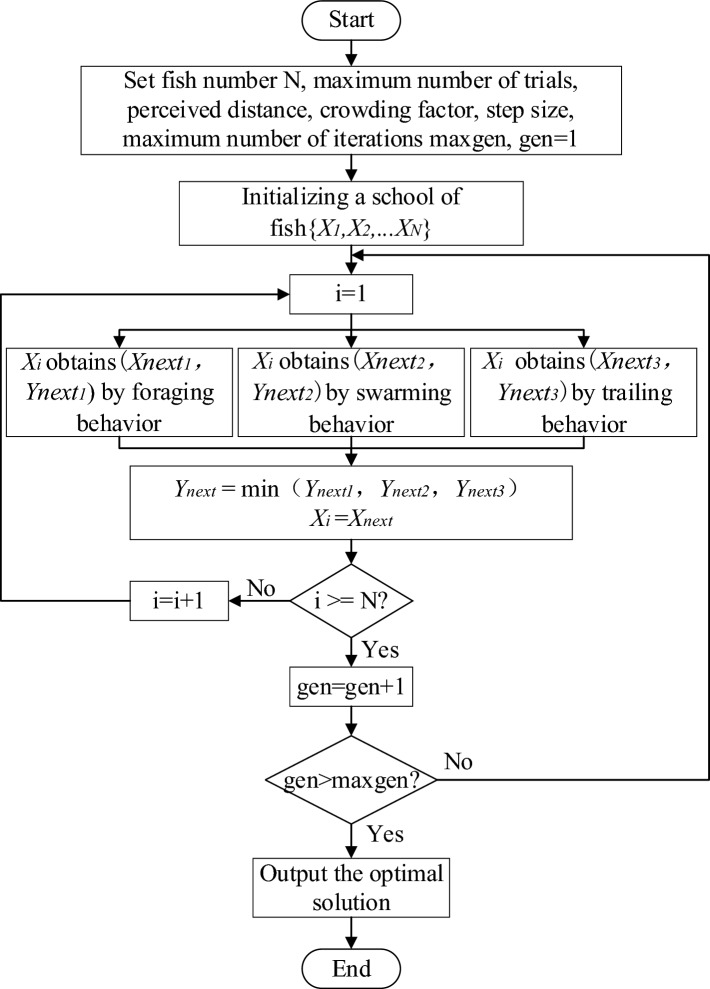
Flowchart of Artificial Fish Swarm algorithm.

**Figure 8:**
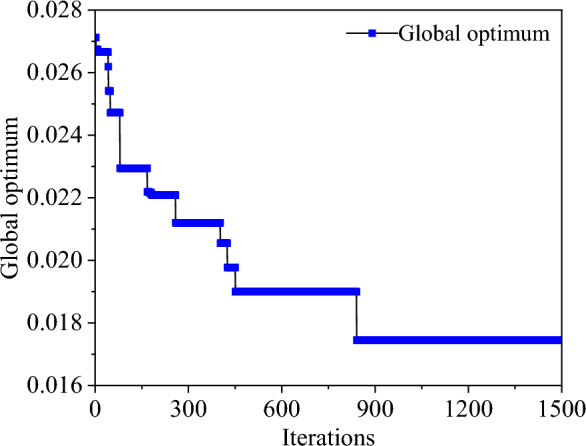
Global optimization process.

Given the global optimization capabilities of the Artificial Fish Swarm algorithm and its ability to avoid issues such as solution dependence on initial values, it is employed to optimize and solve the objective function. During the computation process, to prevent the numerical differences in material parameters from significantly impacting the results, normalization is applied to the four different material parameters. This ensures that all material parameters fall between 0.1 and 0.9. These normalized parameters are then input into the optimization program. After the computation is completed, all material parameter calculation results are reverse-normalized. The computation process is shown in Fig. 7. The fish swarm size is set to 320, the maximum number of probing attempts is set to 4, the perception distance is set to 0.1, the crowding factor is set to 0.618, and the step size is set to 0.05. After the initialized fish swarm undergoes foraging, swarming, and trailing behaviors, the position with the smallest objective function is selected, otherwise, the iteration process is repeated. Once the computation is completed according to the given number of evolution times, the program automatically selects the global optimal solution, which are the material parameters that best match the specimen. For instance, the change in the global optimal value of point 12 during the iteration process is shown in Fig. 8. The objective function reaches convergence after 1500 iterations.

## Result analysis

### Inversion results of displacement field

**Figure 9:**
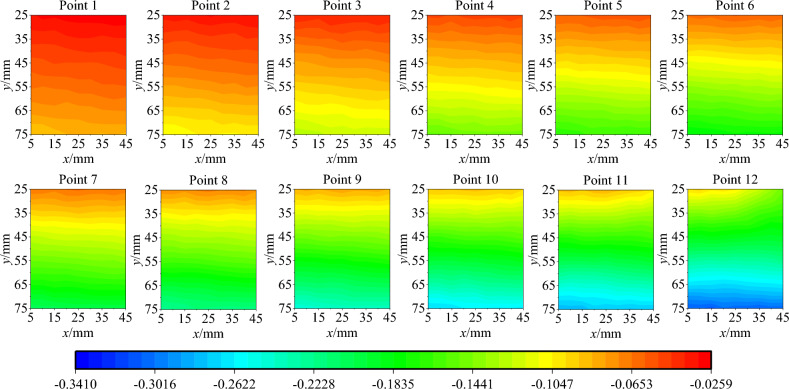
The contour map of the vertical displacement field of test.

**Figure 10:**
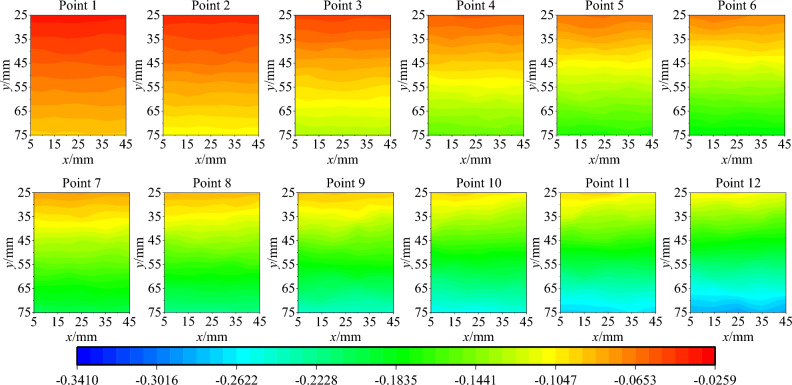
The contour map of the vertical displacement field of inversion results.

**Figure 11:**
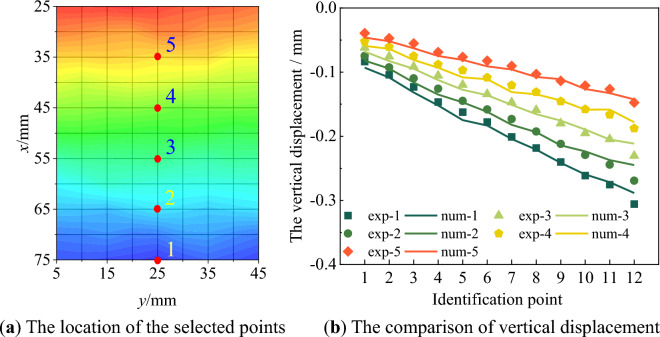
The vertical displacement comparison results of selected points.

Parameter inversion is conducted based on the vertical displacement field data at points 1 to 12. Specifically, the contour maps of the experimental vertical displacement field of points 1 to 12 in the calculation area are shown in Fig. 9, and the contour maps of the vertical displacement field resulting from finite element inversion are depicted in Fig. 10. In particular, to reduce the influence of the upper and lower ends of the specimen on the calculation results, the calculation area is selected as the central part of the speckle surface of the specimen. By comparing Figs. 9 and 10, it can be observed that the distribution characteristics of the vertical displacement field from the finite element inversion result are in agreement with those of the experimental vertical displacement field. Within the calculation area, five points are selected along the vertical direction, as shown in Fig. 11a. The changes in the vertical displacement field of the finite element inversion result and the experimental vertical displacement field during the loading process are calculated respectively, as shown in Fig. 11b. In this figure, 'exp' denotes experimental displacement, and 'num' stands for model displacement. For the elastic and plastic loading stages corresponding to points 1 to 12, the magnitude and trend of the experimental vertical displacement are generally consistent with those of the vertical displacement in the finite element model.

### Inversion results of parameter field

**Figure 12:**
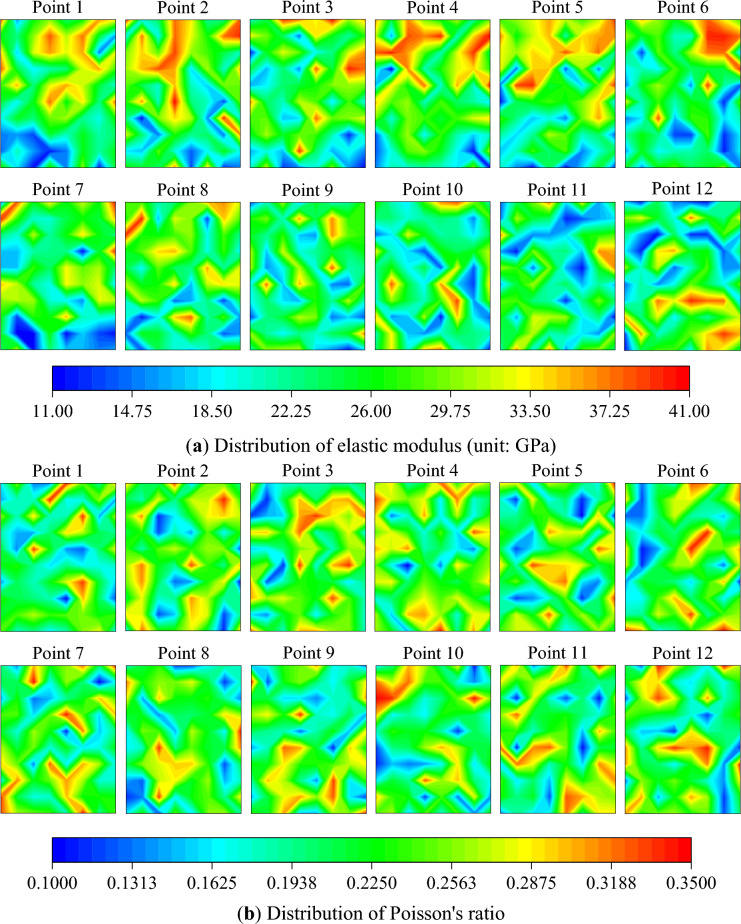

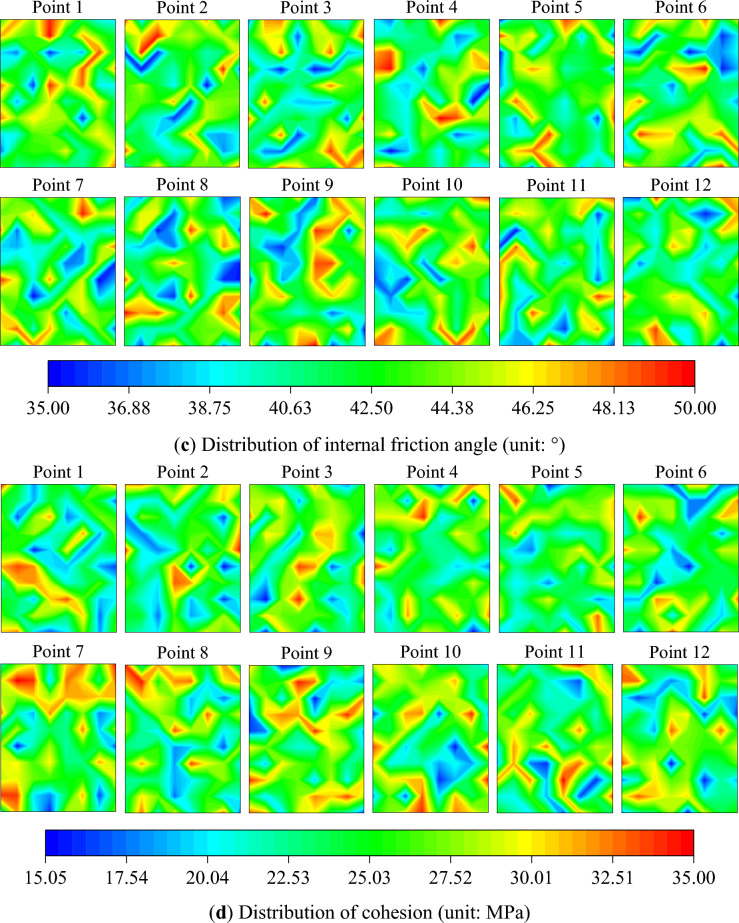
Inversion results of material parameters.

**Figure 13:**
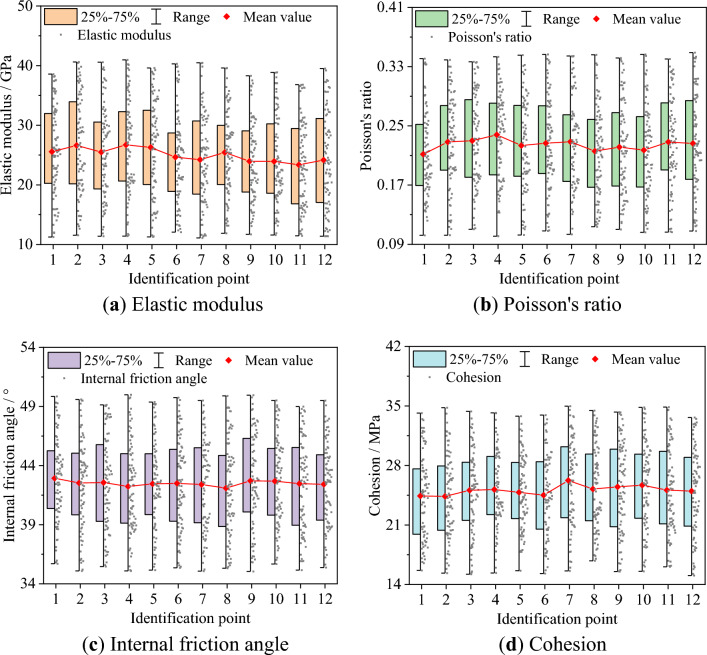
Boxplots of material parameters.

**Figure 14:**
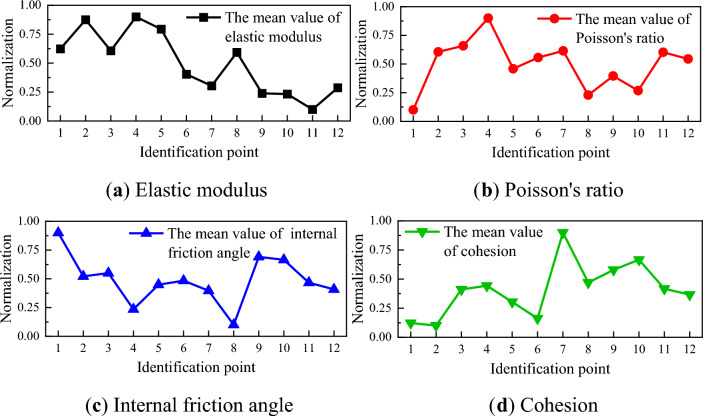
The normalized result of the mean value of material parameters.

The inversion results of the elastic modulus *E*, Poisson's ratio $\mu$, internal friction angle $\varphi$, and cohesion $c$ within the calculation area are presented as contour maps and boxplots, as depicted in Figs. 12 and 13. The mean value of material parameters at different loading times are calculated to understand the overall characteristics of the material parameters. Furthermore, to clearly comprehend the changing trend of the mean value of material parameters during the loading process, the material parameters obtained from the inversion results are normalized, as shown in Fig. 14.

The mean value of the elastic modulus peaks at 26.72 GPa at point 4 and dips to its lowest at 23.37 GPa at point 11. During the elastic stage, the mean value of the elastic modulus experiences minor fluctuations, then decreases upon transitioning into the elastic–plastic stage, and slightly increases in the early failure stage. In the later stages of loading, the mean value of the elastic modulus decreases by 5.51% compared to the initial loading stage. The mean value of Poisson's ratio peaks at 0.2379 at point 4 and dips to its lowest at 0.2117 at point 1. The mean value of Poisson's ratio initially increases then decreases during the elastic stage, decreases upon entering the elastic–plastic stage, and increases in the early failure stage. In the later stages of loading, the mean value of Poisson's ratio increases by 6.89% compared to the initial loading stage. The mean value of the internal friction angle peaks at 42.93° at point 1 and dips to its lowest at 42.09° at point 8. The mean value of the internal friction angle gradually decreases during the elastic stage, increases upon entering the elastic–plastic stage, and slightly decreases in the early failure stage. In the later stages of loading, the mean value of the internal friction angle decreases by 1.20% compared to the initial loading stage. The mean value of the cohesion peaks at 26.24 MPa at point 7 and dips to its lowest at 24.36 MPa at point 2. After slight fluctuations during the elastic stage, the mean value of the cohesion remains essentially unchanged, then gradually decreases upon entering the elastic–plastic stage. In the later stages of loading, the mean value of the cohesion increases by 2.34% compared to the initial loading stage.

The non-uniform parameters obtained from the inversion are fitted to a Weibull distribution. The cumulative distribution function $F\left(a\right)$ and the probability density function $f\left(a\right)$ of the Weibull distribution are as follows:

4 $$F\left(a\right)=1-\mathrm{exp}[-{\left(\frac{a}{{a}_{0}}\right)}^{m}]$$

5 $$f\left(a\right)=\frac{m}{{a}_{0}}{\left(\frac{a}{{a}_{0}}\right)}^{m-1}\mathrm{exp}[{-\left(\frac{a}{{a}_{0}}\right)}^{m}]$$

In the aforementioned equations, *m* is the shape parameter, which signifies the non-uniformity of the material parameters, $\alpha$ represents the material parameters for each element in the model, and ${\alpha }_{0}$ is the mean value of the material parameters. Given the known material parameter $\alpha$, the objective is to solve for the mean value ${\alpha }_{0}$ and the shape parameter *m*. By taking the double logarithm on both sides of the cumulative distribution function $F\left(a\right)$, we obtain:

6 $$\mathrm{ln}\left[-\mathrm{ln}\left(1-F\left(a\right)\right)\right]=m\cdot ln\left(a\right)-m\cdot ln({\alpha }_{0})$$

The equation is rewritten in the form of Y = BX + A:

7 $$\mathrm{Y}=\mathrm{ln}\left[-\mathrm{ln}\left(1-F\left(a\right)\right)\right]$$

8 $$B=m$$

9 $$X=ln\left(a\right)$$

10 $$A=-m\cdot ln({\alpha }_{0})$$

The coefficients are solved using the method of least squares:

11 $$B=\frac{\sum \left({X}_{i}-\overline{X }\right)\left({Y}_{i}-\overline{Y }\right)}{\sum {\left({X}_{i}-\overline{X }\right)}^{2}}$$

12 $$A=\overline{Y }-B\overline{X }$$

13 $$m=B$$

14 $${a}_{0}=\mathrm{exp}[-\frac{(\overline{Y }-B\overline{X })}{m}]$$

**Figure 15:**
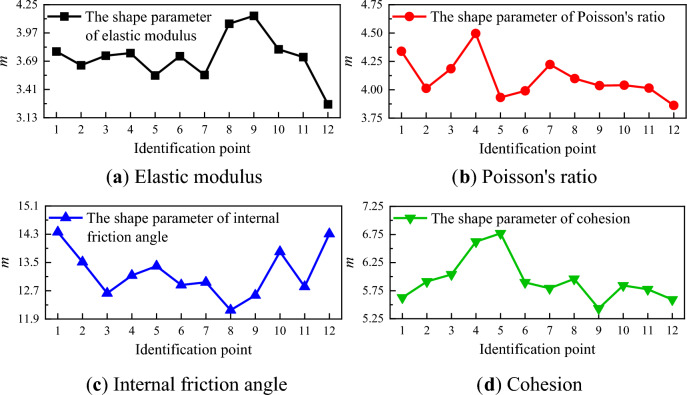
The shape parameter of material parameters for identification points 1–12.

The shape parameter *m*, as derived from the fitting, is depicted in Fig. 15. During the elastic stage, the shape parameter of the elastic modulus remains relatively stable, but increases upon entering the elastic–plastic stage, resulting in a decrease in non-uniformity. Prior to failure, the shape parameter decreases, leading to an increase in non-uniformity. The shape parameter of Poisson's ratio exhibits significant fluctuations during the elastic stage and gradually decreases during the elastic–plastic stage and the period preceding failure, thereby enhancing non-uniformity. The shape parameter of the internal friction angle initially decreases during the elastic stage and then stabilizes, with substantial fluctuations occurring during the elastic–plastic stage and the period before failure, with the general trend being an increase. The shape parameter of cohesion initially increases and then decreases during the elastic stage, decreases during the elastic–plastic stage and the period preceding failure, thereby intensifying non-uniformity.

The points 1 to 12 depicted in Fig. 3 fall within stages 2 and 3. During the elastic stage, the micro-cracks within the specimen undergo rapid closure. The underdeveloped state of these cracks over a short duration does not induce significant changes in rigidity, but it does result in certain elastic deformations within the specimen. Upon transitioning into the elastic–plastic stage, the micro-cracks within the rock specimen undergo rapid development, leading to a slight decrease in the overall strength. Prior to the specimen's failure, the micro-cracks rapidly evolve into macro-cracks, resulting in a reduction in strength at the crack site, an increase in lateral deformation, a decrease in the mean of the elastic modulus, an increase in the mean of Poisson's ratio, and an enhancement in non-uniformity. The mean of the internal friction angle increases upon entering the elastic–plastic stage, while the mean fluctuation of cohesion decreases. This is consistent with the trends in the evolution of the material parameters in the inversion results, and aligns with the conclusion drawn in references^37–39^, which state that during the failure process of rock materials, the internal friction angle gradually increases during the elastic–plastic stage, and the cohesion gradually decreases.

## Conclusions

1. This study presents a novel approach for the inversion of elastic–plastic constitutive material parameters in rock, leveraging the Digital Image Correlation (DIC) and Finite Element Model Updating (FEMU) techniques. This method facilitates the capture of the spatially non-uniform distribution field of rock specimen material parameters. Furthermore, it provides insights into the temporal evolution of these material parameters throughout the loading process.
2. Utilizing uniaxial compression tests, the objective function is defined as the sum of the squares of the differences between the experimentally measured displacements and the numerically simulated displacements in the vertical direction. The Artificial Fish Swarm algorithm is employed to optimize this objective function, thereby deriving the material parameters that best correspond to the test specimen.
3. The material parameters of the red sandstone uniaxial compression specimen are inverted. The distribution characteristics of the vertical displacement field from the finite element inversion results align with those of the experimental vertical displacement. The values and trends of the experimental vertical displacement and the numerical vertical displacement are fundamentally consistent.
4. As microcracks rapidly develop under loading, the mean value of the elastic modulus decreases, and its non-uniformity initially decreases and then increases. The mean value and non-uniformity of Poisson's ratio both increase. The mean value of cohesion decreases, and its non-uniformity increases. The mean value of the internal friction angle increases, and its non-uniformity decreases.

## Data Availability

The datasets used and/or analysed during the current study available from the corresponding author on reasonable request.
